# Quantitative evaluation of an information leaflet to increase prompt help-seeking for gynaecological cancer symptoms

**DOI:** 10.1186/s12889-016-3032-y

**Published:** 2016-05-04

**Authors:** Melanie Morris, Claire Friedemann Smith, Emily Boxell, Jane Wardle, Alice Simon, Jo Waller

**Affiliations:** Health Behaviour Research Centre, University College London, 1-19 Torrington Place, London, WC1E 7HB UK; Present address: Cancer Research UK Cancer Survival Group, London School of Hygiene and Tropical Medicine, London, UK; Present address: Health Services Research, School of Health Sciences, City University London, London, UK

**Keywords:** Gynaecological cancer, Help-seeking, Knowledge, Barriers, Anxiety, Leaflet, Early diagnosis

## Abstract

**Background:**

Provision of written information may improve awareness of cancer symptoms and encourage timely presentation in primary care. This study assessed changes in symptom knowledge, perceived barriers to help-seeking, anxiety and intention to seek help, following exposure to a leaflet to raise awareness of gynaecological cancer symptoms.

**Methods:**

Women (*N* = 484) completed questionnaires before and after reading the leaflet. The primary outcome was change in anticipated time to help-seeking for 12 symptoms. Changes in symptom knowledge, barriers and anxiety, and their association with prompt help-seeking were evaluated using Wilcoxon signed rank tests and logistic regression analyses.

**Results:**

After reading the leaflet, symptom knowledge increased (*p* < 0.001), and perceived barriers (*p* < 0.001) and anxiety (*p* = 0.008) decreased. The number of symptoms for which women anticipated seeking help promptly increased (*p* < 0.001). Changes in knowledge (OR 4.21, 95 % CI 1.95-9.13) and perceived barriers (OR 4.60, 95 % CI 1.91-11.04) were independently associated with increased help-seeking.

**Conclusion:**

Increased symptom knowledge and lowered perceived barriers were related to increased prompt anticipated help-seeking. This occurred without an increase in anxiety. This intervention is effective in altering knowledge, beliefs and help-seeking intentions for gynaecological cancer symptoms, at least in the short-term, and should be trialled in primary care.

**Electronic supplementary material:**

The online version of this article (doi:10.1186/s12889-016-3032-y) contains supplementary material, which is available to authorized users.

## Background

Gynaecological cancers are a significant burden in the United Kingdom (UK): they have a combined incidence second only to breast cancer [1] and incidence is likely to rise as the population ages and becomes more overweight [2]. Some symptoms of gynaecological cancers can be present long before diagnosis [3], suggesting that earlier diagnosis could be made on the basis of these symptoms. However, some of these symptoms are relatively common and have a low positive predictive value [3–5] with the result that they may be mistaken for symptoms of more benign conditions by patients and healthcare professionals alike [6]. Although in 2015 the National Institute for Health and Care Excellence (NICE) lowered the threshold at which patients should be referred for cancer diagnostic tests [7], if the patient herself does not recognise and act upon symptoms there is no opportunity for referral for investigations and early diagnosis.

Due to the progressive nature of cancer, much effort has been put into understanding what contributes to a shorter patient interval – the time taken for an individual to notice and interpret a new symptom as worthy of medical attention and to seek medical advice [8]. Shorter patient intervals could lead to earlier-stage diagnoses and improved survival [9, 10]. Studies of women’s levels of awareness of gynaecological cancer symptoms show that free recall of symptoms is low and, although prompted recognition is higher, most women still do not recognise all symptoms [11–13]. Furthermore some symptoms (e.g., abdominal pain) are well-recognised by women as important, while others (e.g., feeling full quickly and difficulty eating) are less well-known as possible symptoms of ovarian cancer [12]. When women experience these symptoms, they typically attribute them to benign causes rather than cancer [14], and may not seek a medical opinion promptly.

UK studies have reported that participants who do not recognise a symptom as being a cancer alarm symptom are more likely to anticipate waiting longer to seek help than those who do [15, 16]. In addition, when General Practitioners (GPs) were asked what they thought caused delay in presentation for gynaecological cancer symptoms they agreed that patients’ lack of knowledge was a substantial barrier [17]. This is consistent with the finding that knowledge that a symptom could be caused by cancer acts as a prompt to help-seeking [18]. However, there is a dearth of research on interventions to promote prompt help-seeking in this context [19].

The provision of information is an ongoing challenge for healthcare providers. Completeness of information must be balanced with considerations of the target population’s health literacy and the importance of not inducing undue anxiety, which itself can be a barrier to help-seeking [20]. To date, the use of focus groups and other qualitative methods to develop and refine written information has dominated this area, while experimental evaluation of the impact of information leaflets is rare. Qualitative evaluations of health information leaflets tend to report descriptive results about whether the leaflet was found to be acceptable and comprehensible by the patients [21–23], and although this can be an important step in development, how effective written information is at changing attitudes or behaviours is still under-researched [24].

This study therefore represents a crucial, but often-neglected stage of testing in leaflet development. It is an attempt to go beyond the focus groups and interviews [17] that have already informed the initial development of this leaflet, and obtain a quantitative measure of its immediate impact before testing its effect on consultation rates in primary care. This approach is in line with the Complex Interventions Guidance from the Medical Research Council (MRC) which emphasises the need for a ‘development-evaluation-implementation’ process, and for *‘greater investment in developmental studies prior to large-scale evaluations*’ [25] (p33).

In the current study, we evaluated a leaflet designed for use in a primary care setting which aims to increase appropriate presentation of symptoms associated with gynaecological cancers. It does this by laying out the symptoms clearly, reassuring women about their potential concerns and including a checklist of symptoms that might empower them to take their concerns to a healthcare professional. We quantified the change, after reading the leaflet, in symptom knowledge, barriers to presentation and anxiety, and assessed whether these were related to anticipated prompt help-seeking.

## Methods

### Recruitment

We calculated that a sample size of 464 would be required to detect a small effect size of 0.26, based on the changes reported in help-seeking intentions following a leaflet intervention in a previous study [26]. Women were recruited in and around London at ten Cancer Awareness Roadshows and seven Race for Life^1^ events (both runners and spectators). Race for Life events are specifically for women and the Roadshows visit deprived inner-city areas. In this way we reached a range of women who, despite a potential interest in cancer, would not necessarily have any more knowledge about symptoms than the general population. Women over 18 were consecutively invited to take part in the research, including those who had had a hysterectomy or a cancer diagnosis as the leaflet was still relevant for them, until the required sample size was reached. Women were offered fruit and drinks while participating, and given a supermarket trolley token in lieu of a financial incentive. Women read a participant information sheet and signed a consent form after being given the opportunity to ask questions. Participants were given a Cancer Research UK helpline card in case they had any further questions about cancer. Data were collected between April 2011 and August 2011.

### Protocol

Women were approached to complete a questionnaire before (Time 1 – T1) and after (Time 2 – T2) reading the intervention leaflet. Health literacy was assessed before the first questionnaire using a verbal health literacy assessment (Newest Vital Sign – NVS [27]) which has been validated for use in the UK [28]. Demographic information was collected in the second questionnaire. Questionnaires were completed either in a paper and pencil format or on a laptop computer on which an identical questionnaire was laid out. Researchers were trained in the protocol to ensure consistency of administration. The process took approximately 30 min for each participant. Ethical approval was granted by the University College London (UCL) Research Ethics Committee (reference 1122/004).

### Materials

#### Leaflet

The leaflet was previously developed through an iterative process involving consultations with six experts in the field, one-to-one interviews with three gynaecological cancer survivors, and three focus groups (two with non-symptomatic women, one with survivors). The leaflet contains a GP’s message inviting women to come forward with any concerns; introduces the five gynaecological cancers with a diagram of the female reproductive system; and lists the symptoms of the gynaecological cancers. It deals with common concerns, and attempts to reassure women about potential embarrassment or worry. Finally, it includes a symptom checklist on which women could record any symptoms they have had and note when they started. The leaflet ends with a ‘call to action’ for women with any symptoms to call their GP surgery for an appointment (Additional file 1).

#### Questionnaire

The questionnaire measures were derived from the Ovarian Cancer Awareness Measure and the Cervical Cancer Awareness Measure (CAM) [29] with the addition of specific questions about symptoms relevant to the leaflet. Demographic characteristics were assessed using questions from the Office for National Statistics [30]. The questionnaires were piloted with members of the public (*N* = 12) and amended according to comments.

##### Knowledge of symptoms

Women’s recognition of gynaecological cancer symptoms was assessed by presenting a list of 12 symptoms and asking them to indicate which ones were warning signs of gynaecological cancers. All of the symptoms presented were in fact correct symptoms. Women’s symptom knowledge was summed both before and after exposure to the leaflet, giving a range from 0 to 12 at each time point.

A symptom knowledge change score was calculated for each woman by subtracting knowledge scores achieved before the leaflet (T1) from those after the leaflet (T2). This continuous variable was grouped for the logistic regression analyses into three categories: 1) no change or a reduction in knowledge, 2) a small increase in knowledge (one to six symptoms), or 3) a large increase (seven or more symptoms).

##### Barriers

Women’s perceived barriers to help-seeking were also assessed with questions from the CAM [31]. Here the women were presented with a list of 10 potential barriers including four emotional barriers (e.g., embarrassment), three practical barriers (e.g., too busy) and three service barriers (e.g., worried about wasting doctor’s time) [16]. For each barrier they were asked to answer ‘yes often’ (coded as 1), ‘yes sometimes’ (coded 1), ‘no’ (coded 0) or ‘don’t know’ (coded as missing). Scores were summed to give a barrier score (range 0 to 10) at both T1 and T2.

A barrier change score was calculated by subtracting the number of barriers reported at T1 from the number reported at T2. For some analyses this was grouped into three categories: 1) an increase or no change in barriers, 2) a small reduction in barriers (one to two) and 3) a large reduction in barriers (two or more).

##### Anxiety

The short form of the State-Trait Anxiety Inventory (STAI) [32] was used to assess women’s anxiety levels at the start of each questionnaire. The 6 item scale has a range of 6 to 24, with higher scores indicating greater anxiety. Scores were imputed for women who had missing data if they had answered more than half (**>**3) of the items, by calculating a mean score from the completed items. An anxiety change score was calculated by subtracting the STAI score at T1 from the score at T2. These were also grouped into three categories: 1) a large reduction in anxiety (by seven or more points), 2) a small reduction in anxiety (by one to six points), and 3) no change or an increase in anxiety.

##### Anticipated time to help-seeking

Anticipated time to seeking help from the GP was measured using questions derived from the CAM [31]. Initially each woman was asked to indicate how quickly she might go to the doctor for each of the 12 symptoms using ten time categories ranging from ‘1 to 3 days’ to ‘never’. A score was developed from these answers to give a measure of the speed with which each woman anticipated seeking help compared to the other women in the sample. Although previous studies have explored anticipated help-seeking using a somewhat arbitrary cut-off of two weeks [16], the symptoms assessed in this study varied greatly in their urgency and no overall guidance exists for appropriate clinically-meaningful cut-offs. Therefore, for each symptom women were dichotomised into those who intended to seek help by the median-chosen category or sooner (coded as 1), and those who would wait longer than the median (coded as 0). Attending before the median was considered prompt help-seeking, as it was faster than the average in the sample. Scores were then summed across all 12 symptoms (range 0–12) to give a “promptness score” with higher scores indicating prompt help-seeking for a greater number of symptoms.

For responses at T2 the same method was used, but maintaining the medians used at T1 as the cut-offs, so that anticipated time to help-seeking at T2 could be compared with women’s T1 responses. A binary change score was created for use in the logistic regression analysis: women who would seek help promptly for more symptoms at T2 were coded as 1, and those who would seek help promptly for the same number or fewer symptoms at T2 were coded as 0.

##### Demographic characteristics

For the analyses, age was grouped into five categories (18–29, 30–39, 40–49, 50–59, and over 60 years old) and ethnicity was dichotomised into White/non-White. Education level was used as a proxy for socio-economic status with three levels defined: no formal qualifications, school-level qualifications and higher-education qualifications.

##### Health literacy

Health literacy was measured by the NVS (see above), and was grouped into three categories; low (0–1 points), marginal (2–3 points), and adequate literacy (4–6 points).

### Statistical analysis

Changes in reported symptom knowledge, barriers, anxiety and anticipated time to help-seeking from T1 to T2 were evaluated using Wilcoxon signed rank tests. Univariate and multivariate binary logistic regression analyses were performed to measure the extent to which change in knowledge score, change in barriers score, and change in anxiety score were associated with a change in anticipated help-seeking, using the binary outcome variable described above. Age, ethnicity, education, and health literacy were included in all the multivariate models as they are known from previous research to be associated with help-seeking behaviour.

Analyses were carried out using SPSS Version 20.

## Results

### Sample characteristics

A sample of 484 women was recruited. Table 1 shows the demographic characteristics of the sample: 43 % (*n* = 207) were over 40 years old (mean = 41 years, SD = 14 years, range 18 to 83 years). Half had a higher degree (54 %, *n* = 260), and the majority scored in the ‘adequate’ range for health literacy (58 %, *n* = 282). The sample was more ethnically varied than the UK population, with a quarter of women from non-white ethnic minority groups (25 %, *n* = 119). Almost a third of women (32 %, *n* = 148) reported experiencing symptoms in the last three months, but very few participants had had a diagnosis of gynaecological cancer themselves (<3 %, *n* = 12). However 38 % knew someone who had (*n* = 176, data not shown).

**Table 1:** Characteristics of sample (*n* = 484)

	Number	Percent
Age		
18–29	113	23.3
30–39	95	19.6
40–49	94	19.4
50–59	61	12.6
60+	52	10.7
Missing	69	14.3
Ethnicity		
White	348	71.9
Other ethnic backgrounds	119	24.6
Missing	17	3.5
Highest qualification		
No formal qualifications	37	7.6
School education (A levels or GCSEs)	147	30.4
Higher education (degree or above)	260	53.7
Missing	40	8.3
Health Literacy		
Low (0–1)	64	13.2
Marginal (2–3)	120	24.8
Adequate (4–6)	282	58.3
Missing	18	3.7

### T1 responses

At T1, the median time that women anticipated waiting before seeking help varied across symptoms from 1 week (for pelvic/abdominal pain that doesn’t go away, persistent vaginal discharge with an unpleasant odour, soreness or lump on the vulva) up to 6 weeks (for longer of heavier periods than usual) (Table 2). At T1 the mean score for symptom knowledge was 6.6 (SD = 3.3), for barriers endorsed was 3.5 (SD = 2.5), and for anxiety was 12.8 (SD = 4.5).

**Table 2:** Anticipated time to help-seeking for each symptom before and after reading the leaflet

	Median anticipated time to help-seeking		Number (%) of women anticipating *never* seeking help for symptom	
Symptom given in the questionnaire	Time 1 (pre-intervention)	Time 2 (post-intervention)	Time 1 (pre-intervention)	Time 2 (post-intervention)
Pelvic/abdominal pain that doesn’t go away	1 week	4–6 days	11 (2 %)	5 (1 %)
Persistent vaginal discharge with an unpleasant odour	1 week	4–6 days	26 (5 %)	8 (2 %)
Soreness or lump on the vulva (outer part of the vagina)	1 week	4–6 days	42 (9 %)	14 (3 %)
Bleeding after the menopause	2 weeks	1 week	32 (7 %)	14 (3 %)
Vaginal bleeding between periods	2 weeks	1 week	20 (4 %)	8 (2 %)
Lower back pain that doesn’t go away	2 weeks	1 week	31 (6 %)	16 (3 %)
Persistent diarrhoea or other changes in bowel habits	2 weeks	1 week	15 (3 %)	8 (2 %)
Bloating / swollen tummy	1 month	1 week	56 (12 %)	20 (4 %)
Loss of appetite / feeling full quickly	1 month	1 week	81 (17 %)	24 (5 %)
Pain / discomfort during sex	1 month	1 week	41 (9 %)	17 (4 %)
Needing the toilet more often or more urgently	1 month	1 week	25 (5 %)	12 (3 %)
Longer or heavier periods than usual	6 weeks	1 week	45 (9 %)	15 (3 %)

### Changes from T1 to T2

The median time that women would wait before seeking help was reduced after reading the leaflet across all symptoms, with the greatest reduction for “Longer or heavier periods”. The number who anticipated never seeking help for each symptom was reduced by at least half, and up to 77 % for lump or soreness on vulva and longer or heavier periods (Table 2).

Symptom knowledge, barriers, anxiety levels and anticipated time to help-seeking were all significantly different after the intervention. Symptom knowledge increased from a mean of 6.6 symptoms recognised per woman to 10.1 (out of a maximum of 12, *p* < 0.001) (Table 3). Reported barriers to help-seeking reduced from a mean of 3.5 to 2.5 (out of a possible 10, *p* < 0.001). Anxiety reduced slightly from a score of 12.8 to 12.0 (range 6 to 24, *p* = 0.008).

**Table 3:** Unadjusted mean scores before and after the intervention (only respondents at T1 and T2 included)

	N	Time 1 (Pre-intervention) Mean (SD)	Time 2 (Post-intervention) Mean (SD)	Wilcoxon Signed Rank Test: *p* value
Symptom knowledge^a^	483	6.56 (3.30)	10.10 (3.27)	*p* < 0.001
Barriers to help-seeking^b^	472	3.46 (2.51)	2.46 (2.51)	*p* < 0.001
Anxiety score^c^	401	12.83 (4.50)	12.01 (4.48)	*p* = 0.008
Anticipated help-seeking^d^	484	6.82 (4.15)	8.75 (3.95)	*p* < 0.001

^a^Number of symptoms recognised: range of scores 0–12

^b^Number of barriers endorsed: range of scores 0–10

^c^Short version STAI: range of scores 6–24

^d^Number of symptoms for which the woman would go to the doctor more quickly than the Time 1 median

Before reading the leaflet, 21 % of women anticipated seeking help for every one of the 12 symptoms quicker than the median category chosen (i.e., they had a promptness score of 12). However after reading the leaflet, that proportion doubled to 43 %, and the proportion who did not anticipate seeking help quicker than the median for any symptom (promptness score = 0) also decreased (from 10 to 7 %). The mean number of symptoms for which women would seek help more quickly than the median increased from 6.8 symptoms before exposure to the leaflet, to 8.8 symptoms after (*p* < 0.001).

### Binary logistic regression analysis

In univariate analyses (Table 4), an increase in the number of symptoms for which women would seek help promptly was significantly associated with being more highly educated (OR 2.59, 95 % CI 1.29–5.53), an adequate health literacy score (OR 1.77, 95 % CI 1.01–3.10), both a small increase (OR 2.55, 95 % CI 1.54–4.20) and a large increase (OR 4.63, 95 % CI 2.62–8.21) in the participants’ symptom knowledge, and both a small and a large reduction in the number of barriers endorsed (OR 1.82, 95 % CI 1.22–2.73 and OR 3.91, 95 % CI 2.08–7.34 respectively). Conversely, being of non-white ethnicity was associated with a reduced likelihood of prompt help-seeking (OR 0.64, 95 % CI 0.42–0.98).

**Table 4:** Predictors of prompt anticipated help-seeking for more symptoms at Time 2 than Time 1 (excludes missing data)

		Univariate logistic regression (unadjusted)		Multivariate logistic regression (adjusted)	
	Increase in prompt help-seeking score at T2, n (row %)	Odds ratio	95 % CI	Odds Ratio	95 % CI
Age					
18–29 (113, 27 %)	76 (68 %)	1.00		1.00	
30–39 (95, 23 %)	55 (60 %)	0.70	0.40–1.25	0.88	0.44–1.75
40–49 (94, 23 %)	57 (61 %)	0.73	0.41–1.29	1.00	0.49–2.02
50–59 (61, 15 %)	34 (57 %)	0.62	0.33–1.18	0.71	0.31–1.61
60+ (52, 12 %)	27 (53 %)	0.53	0.27–1.05	0.65	0.24–1.79
Ethnicity					
White (348, 75 %)	215 (63 %)	1.00		1.00	
Non-white (119, 25 %)	60 (52 %)	**0.64**	**0.42–0.98**	**0.54**	**0.29–0.98**
Education Qualifications					
None (37, 8 %)	16 (43 %)	1.00		1.00	
School education (147, 33 %)	79 (55 %)	1.57	0.76–3.25	1.00	0.34–2.90
Higher education (260, 59 %)	170 (66 %)	**2.59**	**1.29–5.53**	1.67	0.57–4.84
Health literacy score					
Low (64, 14 %)	30 (50 %)	1.00		1.00	
Marginal (120, 26 %)	62 (53 %)	1.13	0.61–2.10	1.08	0.38–3.10
Adequate (282, 60 %)	175 (64 %)	**1.77**	**1.01–3.10**	0.94	0.33–2.72
Symptom knowledge change score					
No change or decrease (100, 21 %)	33 (37 %)	1.00		1.00	
Small increase (242, 50 %)	144 (60 %)	**2.55**	**1.54–4.20**	**2.68**	**1.32–5.44**
Large increase (141, 29 %)	101 (73 %)	**4.63**	**2.62–8.21**	**4.21**	**1.95–9.13**
Barrier change score					
No change or increase (212, 45 %)	104 (49 %)	1.00		1.00	
Small reduction (188, 40 %)	117 (64 %)	**1.82**	**1.22–2.73**	**1.84**	**1.07–3.17**
Large reduction (72, 15 %)	57 (79 %)	**3.91**	**2.08–7.34**	**4.60**	**1.91–11.04**
Anxiety change score					
No change or increase (234, 58 %)	138 (61 %)	1.00		1.00	
Small reduction (128, 32 %)	87 (68 %)	2.02	0.97–4.18	1.95	0.82–4.61
Large reduction (39, 10 %)	20 (51 %)	1.46	0.74–2.88	1.39	0.63–3.07

Results in bold are significant (*p* < 0.05)

After adjustment for the other variables in the model (Table 4), non-white women were still around half as likely as white women to seek help promptly for more symptoms (OR 0.54, 95 % CI 0.29–0.98). Reductions found in anxiety scores led to more prompt help-seeking than when anxiety stayed the same or increased, but these associations were not significant. However, positive changes in symptom knowledge and in barriers were both significantly associated with large increased odds of prompt help-seeking after adjustment: a small increase (OR 2.68, 95 % CI 1.32–5.44) or a large increase in knowledge (OR 4.21, 95 % CI 1.95-9.13), as well as a small reduction (OR 1.84, 95 % CI 1.07–3.17), or a large reduction in barriers score (OR 4.60, 95 % CI 1.91–11.04) were all associated with prompt anticipated help-seeking for more symptoms.

## Discussion

This quantitative approach to the evaluation of an information leaflet for gynaecological cancer demonstrated a short-term positive impact of the leaflet on symptom knowledge, perceived barriers and anxiety levels. It also reduced the median time the participants would wait before seeking help across all 12 symptoms. Furthermore, we have shown that this increase in symptom knowledge, and decrease in the number of barriers endorsed, were independently predictive of an increase in the number of symptoms for which these women would seek help promptly. Similar to previous research into information provision on testicular cancer [33], the inclusion of reassuring messages and a symptom checklist which gives women a clear course of action facilitated the uptake of the information while avoiding increased anxiety.

Providing women with knowledge of gynaecological cancer symptoms while addressing barriers to help-seeking is important to improve rates of early diagnosis. This is particularly true because the anatomical location of these types of cancer could mean that embarrassment and unwillingness to seek help may present more significant barriers than for other cancers. This may disproportionately impact women from Black, Asian and Minority Ethnic backgrounds [34], which may in part explain the persistent reticence shown by the non-white women in our study to anticipate seeking help. As education levels and health literacy were adjusted for, these cannot explain the large difference found for this group. Further research should investigate what might be added to or changed in such a leaflet to tackle the barriers to prompt help-seeking that are more pronounced in non-white women.

In addition to embarrassment, the non-specific nature of many symptoms of gynaecological cancers, for example bloating and change in bowel habits, has been found to be a barrier to prompt help seeking as women do not associate such symptoms with gynaecological cancers [14], and are less concerned about symptoms that do not specifically involve the reproductive organs [13]. This study showed that by simply highlighting these symptoms to women, they may be more willing to seek help when they notice these changes.

Our previous research with GPs showed that while they believed the leaflet was important, they were concerned about a potential increase in anxiety and consultation rates [17]. We found that the leaflet was able to reduce the anxiety scores of our participants. This effect was however not associated with an increase in intentions to seek help promptly. While a decrease in anxiety score is a positive effect as previous research has linked high levels of fear and anxiety about cancer to delays in help-seeking [35], the change in the mean anxiety score, although significant, was relatively small and as such may not have been big enough to affect help-seeking intentions. The ways of reducing anxiety and its effect on prompt help-seeking warrant further investigation.

The main strength of this study is that it presents a frequently-lacking quantitative evaluation of a low intensity intervention. Our study also benefitted from an ethnically diverse sample (25 % non-white compared to 14 % in the general population [36]), with a wide age range, and adequate power to produce robust findings. Additionally, using a repeated measures design allowed us to measure changes over time, which showed large differences prospectively, albeit over a short time interval.

While strengthening the study and allowing us to assess changes over time, the test-retest design is also a potential weakness in that some of the effect seen on knowledge could have been the result of the women being more practised at the questions on the second completion of the questionnaire. A longer time before re-test was unfortunately not practical here, but despite this the results do provide a first, objective evaluation of the impact of the information provided. In addition, women may have been motivated to provide responses at T2 that were consistent with researchers’ expectations of change (social desirability bias), particularly in relation to speed of help-seeking. As we did not measure social desirability, we cannot rule this out as a possibility.

The large changes in knowledge and barriers led to very large changes in anticipated help-seeking, with evidence of a ‘dose response’ relationship: larger changes in knowledge and barriers leading to larger changes in promptness of help-seeking. This suggests that the more difference we can make to these factors, the more impact there will be on help-seeking. Therefore, although this study can only demonstrate the short term impact, the large effect found implies great potential to make a longer term difference.

This study sample over-represented higher educational levels compared to the general population (54 % had a higher degree compared to 30 % of the general population [37]) and was recruited from a population with an interest in or connection to cancer because recruitment took place at Cancer Research UK events. Those with higher socio-economic status, implied by the higher education levels, are a group more likely to take part in surveys [38], and so generalising must be done with caution. Despite this, there was a good spread of health literacy scores, which was comparable to that found in other studies [39, 40]. Furthermore, there is no reason to think that the women came to these events with any more knowledge of gynaecological cancers than other members of the public, as in this sample knowing someone with cancer was not found to be associated with increased knowledge of these symptoms (mean symptom knowledge score at T1 was 6.19 in those with no cancer experience (*n* = 42) and 6.62 in those with a friend or family member who had had cancer (*n* = 429); *p* = 0.42).

Although these data were collected in 2011, there is little reason to believe that knowledge of symptoms or other barriers have changed substantially in the intervening time. Recent studies continue to find that women frequently misattribute the symptoms of gynaecological cancer, and that their help-seeking is influenced by barriers such as the demands of everyday life, or the anxiety that surrounds cancer [14, 41]. The importance of exploring and addressing these factors in help-seeking behaviour remains undiminished and we believe our findings make a significant contribution.

Finally, this stage of testing involved measuring intentions only, not actual behaviour, however it has been found that the correlation between intentions and behaviour in prospective studies is 0.53, which could be considered a large effect size based on Cohen’s interpretation of correlations [42, 43]. This part of the development, therefore, constituted a proof of concept, to support the next stage of testing and development in a primary care setting, assessing the impact of the leaflet on consultation rates (particularly in ethnic minority and socioeconomically deprived groups), investigations ordered and referrals made.

## Conclusions

When developing written information aimed at changing behaviour, it is essential to carry out thorough testing in the target population to demonstrate the impact of the intervention on behavioural intention, and to understand the mechanism for that impact. This study shows that useful information can be gathered through the under-used step of quantitative evaluation of a leaflet and as such indicates a method through which more effective patient information documents could be created. Improving rates of early diagnosis of cancer in the UK depends, in part, on ensuring the patient interval is kept as brief as possible. This study has shown that, as a minimum, intentions to seek help for gynaecological cancer symptoms can be improved through information leaflets which also address barriers to help-seeking and provide a tool with which the woman can approach her GP.

Efforts should now be focussed on trialling the leaflet in primary care settings and assessing its impact on help-seeking behaviours in the real world.

### Ethics approval and consent to participate

We confirm all patient/personal identifiers have been removed or disguised so the patient/person(s) described are not identifiable and cannot be identified through the details of the story. All named authors have agreed to the submission and have contributed significantly to the manuscript, and it is not under consideration elsewhere. Ethical approval was granted by the UCL Research Ethics Committee (reference 1122/004), and we have observed appropriate ethical guidelines and legislation in conducting the study described in this paper.

## Additional file

Additional file 1: Gynaecological Cancer Information Leaflet. (PDF 173 kb)

## Abbreviations

CAM: Cancer Awareness Measure
GP: General Practitioner
MRC: Medical Research Council
NAEDI: National Awareness and Early Detection Initiative
NICE: National Institute for Health and Care Excellence
NVS: Newest Vital Sign
STAI: State-Trait Anxiety Inventory
T1: Time 1
T2: Time 2
UCL: University College London
UK: United Kingdom

## Footnotes

1 More information can be found http://www.cancerresearchuk.org/health-professional/prevention-and-awareness/cancer-awareness-roadshow and http://raceforlife.cancerresearchuk.org/index.html
