# Supplementary material for: Quantitative evaluation of an information leaflet to increase prompt help-seeking for gynaecological cancer symptoms
Source: BMC Public Health. 2016 May 4;16:374. doi: 10.1186/s12889-016-3032-y (PMC4855769; doi:10.1186/s12889-016-3032-y)
Supplement: Additional file 1: — Gynaecological Cancer Information Leaflet. (PDF 173 kb) [file 12889_2016_3032_MOESM1_ESM.pdf]

Do you have any of these symptoms?

If the answer is **yes** to even **one** of these symptoms, it is important to make an appointment with your GP **today**.

It might help you to fill out this checklist and bring it to your appointment. This will make it easier to give your GP as much detail as possible on your symptoms.

Symptom	Tick if you have symptom	When did it start?
Abdominal or pelvic pain		
Bleeding between periods, after sex or after the menopause		
Vaginal discharge that is smelly or blood stained		
Longer or heavier periods		
Bloating, gas or constipation that doesn't go away		
Pain during sex		
Lower back pain that doesn't go away		
Pain, lump, ulcer, soreness or persistant itching of the vulva		
Needing to go to the toilet more urgently or frequently than normal		
Feeling full quickly or loss of appetite		

Make a promise to yourself:

If I have **any** of these symptoms I will make an appointment with my GP **today**.

Call our surgery on: 0207 xxx xxxx

Today's date ______________________________

Date and time of your appointment ______________________________

____

Common concerns

- Even if you don't feel ill, you should have your symptoms checked.
- These symptoms can seem mild and unimportant, but if you have any of these symptoms, it is important that you see your GP.
- You might think that your symptoms will just clear up on their own but it **is better to see your GP.**
- Even if you have been to your pharmacist or GP already, **you should visit them again if your symptoms haven't gone away.**

It is likely that these symptoms are not due to cancer, but it is important to have them checked.

You can find more information about gynaecological cancers at:

www.cancerhelp.org.uk

Or by calling **0808 800 4040** (Mon-Fri 9am-5pm) to speak to a Cancer Research UK nurse

LOGO HERE

A MESSAGE FROM YOUR GP

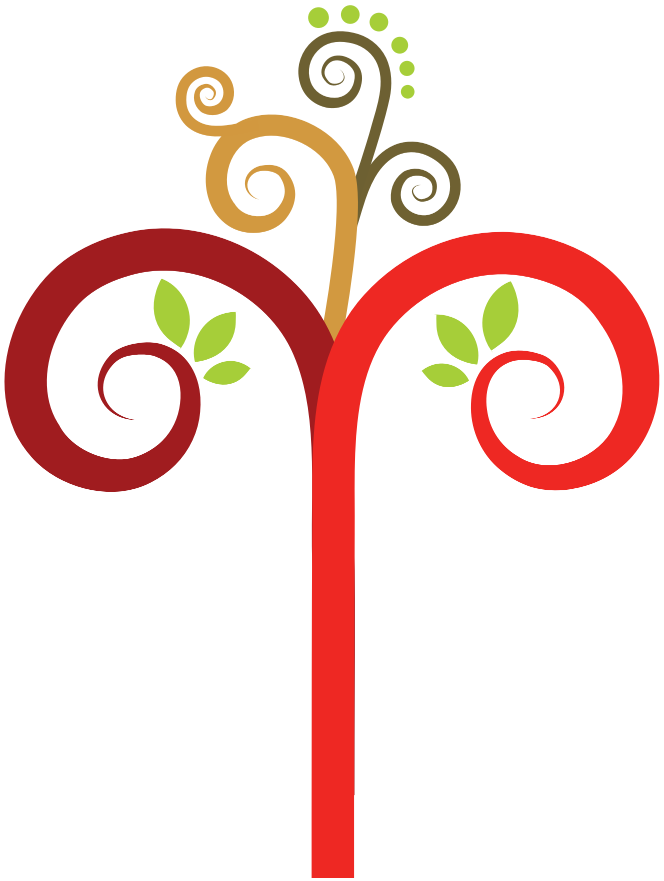

GYNAECOLOGICAL CANCERS

know the signs

Information for Women

Camden NHS
Primary Care Trust

Dear Patient,

I'm sending this leaflet to all women in my practice because I think that it is important that everyone knows about the symptoms of gynaecological cancers.

Every year around 18,000 women of all ages in the UK are diagnosed with a gynaecological cancer.

*I want you to feel able to come to see me if you notice anything that is **not normal for you**.*

*In this leaflet you will find a **symptom checklist** that will help you to see if you need to make an appointment.*

*If you are experiencing **any** of these symptoms please don't delay. **Come to see me**.*

*If you are worried about gynaecological cancer, please speak up. **You will not be wasting my time**.*

Yours sincerely,

(GP's signature)

Dr. Xxxxxxxxxxxx

What are gynaecological cancers?

A gynaecological cancer is a cancer that occurs in your reproductive system:

- Uterus (womb) including endometrium (lining of womb)
- Cervix (neck of womb)
- Ovaries
- Vagina
- Vulva (outside part of the vagina)

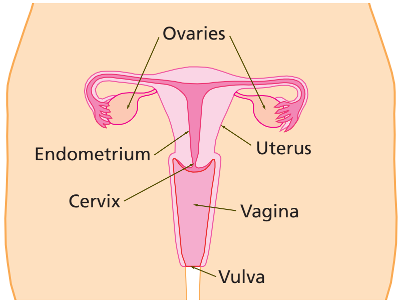

Early signs of gynaecological cancers

The most common symptoms of gynaecological cancers are:

- Abdominal or pelvic pain
- Bleeding between periods, after sex or after the menopause
- Vaginal discharge that is smelly or bloodstained
- Longer or heavier periods
- Bloating, gas or constipation that doesn't go away
- Pain during sex
- Lower back pain that doesn't go away
- Pain, lump, ulcer, soreness or persistent itching of the vulva
- Needing to go to the toilet more urgently and frequently than normal
- Feeling full quickly or loss of appetite

These are important if they are new for you or if they don't go away.

If you are embarrassed or worried...

- You can ask to see a female doctor or ask to have a female member of staff accompany you to your appointment.
- Remember, your GP is used to dealing with sensitive issues and can support you.
- It can help to discuss your concerns with someone you trust among your friends or family.
- You can discuss with your GP any tests that may be offered to you before you choose whether to have them.

The earlier cancer is diagnosed, the better the chances of survival.

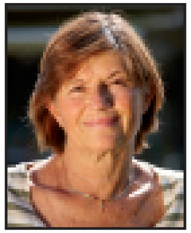

"Cervical cancer never crossed my mind. I was really fit and healthy. I thought people like me didn't get cancer."

Sandra, 64, cervical cancer survivor

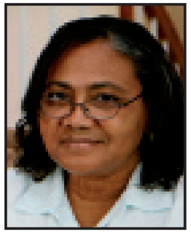

"I thought a normal smear test meant that I was ok. I didn't realise that you could get cancer in other parts."

Meera, 51, ovarian cancer survivor
